# *Fly-LeNet:* A deep learning-based framework for converting multilingual braille images

**DOI:** 10.1016/j.heliyon.2024.e26155

**Published:** 2024-02-14

**Authors:** Abdulmalik Al-Salman, Amani AlSalman

**Affiliations:** a Computer Science Department, King Saud University, Riyadh, Saudi Arabia; b Department of Special Education, King Saud University, Riyadh, Saudi Arabia

**Keywords:** Braille, Deep learning (DL), Multilingual braille images

## Abstract

For many years, braille-assistive technologies have aided blind individuals in reading, writing, learning, and communicating with sighted individuals. These technologies have been instrumental in promoting inclusivity and breaking down communication barriers in the lives of blind people. One of these technologies is the Optical Braille Recognition (OBR) system, which facilitates communication between sighted and blind individuals. However, current OBR systems have a gap in their ability to convert braille documents into multilingual texts, making it challenging for sighted individuals to learn braille for self-learning-based uses. To address this gap, we recommend a segmentation and deep learning-based approach named *Fly-LeNet* that converts braille images into multilingual texts. The approach includes image acquisition, preprocessing, and segmentation using the Mayfly optimization approach with a thresholding method and a braille multilingual mapping step. It uses a deep learning model, LeNet-5, that recognizes braille cells. We evaluated the performance of the *Fly-LeNet* through several experiments on two datasets of braille images. Dataset-1 consists of 1404 labeled samples of 27 braille signs demonstrating the alphabet letters, while Dataset-2 comprises 5420 labeled samples of 37 braille symbols representing alphabets, numbers, and punctuations, among which we used 2000 samples for cross-validation. The suggested model achieved a high classification accuracy of 99.77% and 99.80% on the test sets of the first and second datasets, respectively. The results demonstrate the potential of *Fly-LeNet* for multilingual braille transformation, enabling effective communication with sighted individuals.

## 1 Introduction

There exist 285 million people in the world who have a visual impairment, as reported by the World Health Organization (WHO) [1]. The prevalence of blindness is increasing immensely due to several reasons, such as eye injury, cataracts, amblyopia, etc. Although several braille images have been built to support them, proficiency is low. Moreover, in the United Kingdom, a developed country, the literacy rate of blind people is 4% due to lack of resources [2]. Recently, electronic books have been developed, but the available conversion tool still cannot convert STEM content (science, technology, engineering, and mathematics) into the local language of visually impaired persons. Therefore, much work is still required to transform the content into most local languages that can be used worldwide. Blind persons read the braille systems through fingers tracing the dots, whereas sighted persons can view it via their eyes.

The braille conversion method comprises cells containing six dots that exhibit meaning. Many visually impaired people have used braille systems and found it very useful to communicate with others. On the other side, it is also important for sighted people to understand the braille system quickly and help the visually impaired. In the medical domain, the information is available in printed form in the braille system; however, not all visually compromised people can access and utilize it. Therefore, an automated system is necessary to overcome the challenges mentioned above. The automated systems would also benefit students in schools or colleges [3].

Some traditional systems are based on optical character recognition (OCR) [4] due to the presence of dots in characters rather than the continuous strokes in natural languages [5]. The procedure to capture and process braille documents and transform them into natural language is called an Optical Braille Recognition (OBR) system. The OBR systems mainly comprise three stages: 1) capture images, 2) segment, and 3) text representation. Previously, some investigators acquired braille images through scanners. However, it could not overcome the distortions such as stains on the paper [6]. Thus, in this paper, we propose a DL-based model for a multilingual OBR mechanism, as the existing techniques work primarily for a single language, requiring improvements to be more precise.

The main offerings of the paper are below:

• To propose an automated system named *Fly-LeNet* that can take input of braille images and generate natural language text.
• Our proposed system is easy for visually impaired people to adapt due to its less complex structure.
• We utilized the Mayfly optimization method and thresholding for segmenting the braille cells, which provides the best region of interest covering the braille dots only.
• For recognition, we used the DL model LeNet-5, which is simple and easy to use without requiring high computational systems.
• We trained our model using two datasets and performed various experiments that show the significance of our proposed technique.

## 2 Related work

Several researchers have proposed OBR systems to help visually impaired people [5]. However, these methods have become outdated and may not be accessible worldwide. Each natural language has a braille system. Murray et al. developed a technique for braille documents that had low quality, utilizing the focus of scanning on the smallest part of the document [10]. However, the proposed strategy was slow. Moreover, scanners are feasible for thin and flat paper surfaces, which is not the scenario for braille documents. Therefore, portable devices, including cameras and smartphones, are recommended for capturing an image.

Many approaches are based on image segmentation, which employs an adaptive thresholding algorithm to find the region of interest (ROI), the braille verso and recto dots using the combination rules [7,8]. This segmentation method was sensitive to the threshold value, and the results were attained after numerous steps. Manual feature extraction methods have been used to identify the braille dots to overcome the abovementioned issues. The traditional ML techniques include a histogram of gradient (HOG) and support vector machine (SVM) [9], local binary pattern (LBP) [10], and Haar and SVM [9]. Morgavi et al. utilized a CNN to identify the braille dots [11]. The Hough transform approach has been used to detect braille dots based on a circle.

In recent years, deep learning techniques have been used to develop OBR systems and have attained considerable outcomes due to their automated feature extraction process. Shokat et al. [12] briefly explained various OBR methods in their work. Li et al. [13] developed a segmentation-based model, BraUNet, to recognize braille dots using the encoder-decoder technique. They employed a small dataset; however, the model attained a 99.66% F-score. The dataset was split into three sets: training, testing, and validation. The training set consisted of 74 images, which is a very small set. Kawabe et al. [14] developed a technique identifying the Japanese braille method required to transform old books into electronic books. The technique comprised the Caffe framework and AlexNet network. The model attained between 98% and 99% accuracy for several implementations. However, the model did not achieve significant performance for cell recognition. The braille character segmentation was employed utilizing SVM and HOG feature descriptor to translate English braille alphabets [15]. Primarily, HOG is used for image classification and object detection tasks. Parera et al. [16] developed another SVM technique, adding an extra preprocessing step like contrast enhancement and noise removal. The model attained 96% accuracy; however, the proposed method only worked for scanned images.

Kumar et al. proposed another technique in which input characters were taken from scanned files. The characters then transformed into the braille system. Further, blank spaces were removed, and segmentation was employed. After that, the output was compared with the braille database, and characters became elevated on the pad [17]. Singh et al. developed a system for braille translation from Hindi and English languages using a lookup table [18]. The translation system from math numbers to speech made learning easy for visually impaired people. The system helped non-native speakers who were visually compromised to resolve arithmetic equations easily [[19], [20], [21]].

Braille was transformed in several languages, such as Arabic [22], Hindi [18,23], Urdu [24,25], Tamil [26], Kannada [27], Chinese [28,29], Gujrati [30,31], Sinhala [16], and ODIA [32]. Sana et al. [33] proposed a system for converting braille to English character identification. She used K-nearest Neighbor (KNN), Decision Trees (DT) along with Reconstruction Independent Component Analysis (RICA), SVM, and Principal Component Analysis (PCA) to extract and select features. The maximum accuracy authors attained was 99.79%. The model attained significant results but did not give satisfactory results for mobile devices.

It is true that current techniques based on deep learning models [34] and other methods still have limitations in effectively converting braille images into multilingual texts or voices. One of the main limitations is the inefficiency in transforming wide lengths of braille caused by line-by-line transformation. This means the models must process each line of the braille image separately, which can be time-consuming and unsuitable for large documents. Another limitation is the lack of image segmentation [35] with DL methods to split the braille image page into several lines. This can result in errors and inaccuracies in the text conversion process, especially when dealing with complex layouts. To address these limitations, a more effective method is proposed, which integrates image segmentation with a DL technique to transform braille images into their equivalent multilingual text. This method can improve the accuracy and efficacy of the conversion process.

## 3 Methodology

**Fig. 1:**
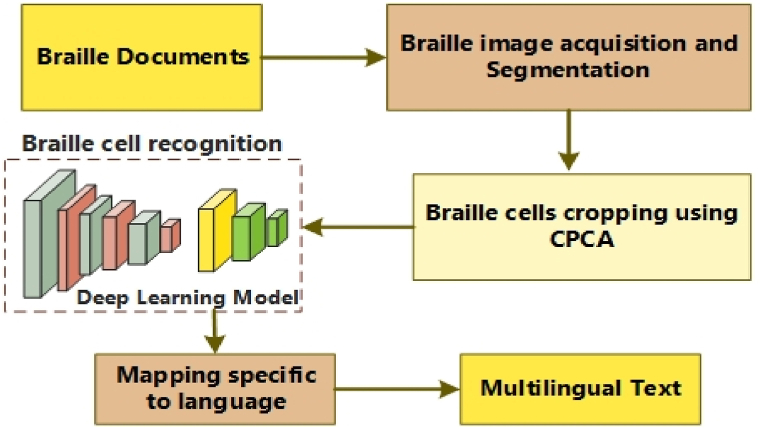
Flow diagram of the *Fly-LeNet*.

The proposed approach for converting braille images into multilingual text involves several sequential steps, as shown in a high-level flowchart in Fig. 1.

The primary step is image acquisition, where the braille sample is acquired as an input. The input sample is preprocessed utilizing image-processing techniques, which may involve techniques such as image enhancement, noise reduction, and image normalization. The outcome of this phase is a preprocessed sample containing a segmented region, which is then passed to the next phase. The second stage is braille cell cropping, where the preprocessed image is divided into individual braille cells. This step involves using image segmentation techniques to identify the boundaries of each braille cell. The output of this phase is a set of cropped braille cell images. The third step is braille cell recognition, where each cropped braille cell image is processed using a deep learning-based character recognition algorithm to identify the corresponding braille character. The output of this step is a set of recognized braille characters. The final step is braille multilingual mapping, where the recognized braille characters are mapped to their corresponding multilingual text using a mapping table. The outcome of this step is the final multilingual text that can be read by a sighted person or utilized by text-to-speech conversions. Overall, the proposed approach combines image processing and segmentation, a deep learning model, and multilingual mapping techniques to enhance the accuracy and efficacy of converting braille images into multilingual text. Algorithm 1 shows the suggested system.

**Figure:**
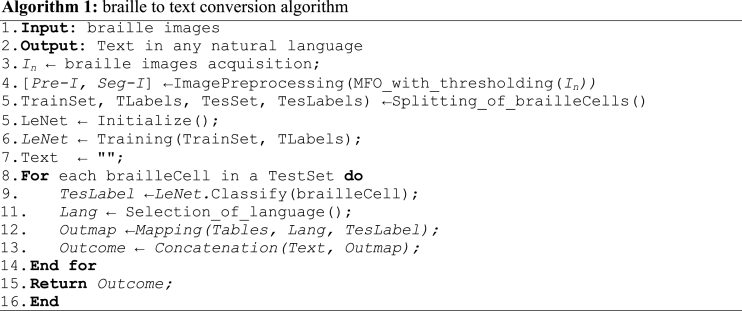

### 3.1 Image acquisition and segmentation

In the first step, the braille samples are gathered utilizing various devices like scanners, cameras, or digital cameras. Image preprocessing is an essential step in braille recognition as it helps to enhance the quality of the input images before they are processed by the deep learning-based model. The preprocessing step involves several sub-steps, such as image segmentation and correcting any skewing or misalignment in the image. We utilized a multilevel thresholding method with the mayfly optimization (MFO) [33] algorithm in this particular approach. The aim is to select the optimal threshold.

Kapur et al. [36] suggested a threshold-based method to calculate the best threshold to segment the region of interest. The method exhibits the best threshold by maximizing the entropy. An objective computation method can be achieved for the calculation of the bi-level threshold, as presented in Eq. (1).

(1) $${FUN}_{kap}\left(t\right)=k_{1}+k_{2}\text{,}$$

The ${FUN}_{kap}\left(t\right)$ is used for evaluation, and *t* refers to the threshold value that exploits the evaluation function. Here, $k_{1}$ and $k_{2}$ are computed as in Eqs. (2), (3)):

(2) $$k_{1}=\sum_{s=1}^{t}\frac{p_{s}}{\omega_{0}}\ln\left(\frac{p_{s}}{\omega_{0}}\right)\text{,}$$

(3) $$k_{2}=\sum_{s=t+1}^{L}\frac{p_{s}}{\omega_{1}}\ln\left(\frac{p_{s}}{\omega_{1}}\right)\text{,}$$

Here, $p_{s}$ demonstrates the probability distribution (PD) of the gray intensity level, and *L* is the no. of gray levels. For multilevel thresholding, the entropy-based method is useful. It is important to divide the braille samples into *n*-class labels employing *n* − 1 threshold values to obtain braille cells. The objective numbers are changed, as exhibited in Eq. (4).

(4) $${FUN}_{kap}\left(T\right)=\sum_{s=1}^{n}k_{s}\text{,}$$

Here, *T =* [*t*_1_, *t*_2_, *t*_(*n*−1)_] refers to a vector comprising multiple threshold values. The entropies are demonstrated distinctly in accordance with threshold *t*. Consequently, Eq. (5) is given for *n* entropy as below.

(5) $$k_{n}^{c}=\sum_{i=t_{n+1}}^{L}\frac{p_{i}}{\omega_{n-1}}\ln\left(\frac{p_{i}}{\omega_{n-1}}\right)\text{,}$$

where, ($\omega_{0}^{c},\omega_{1},\ldots .,\omega_{n-1})$ demonstrates the probability of frequency of the n classes, and for obtaining the best threshold values, the MFO technique is employed. The task of MFO was to search the histogram and find the best threshold in the range of {0 − *L-*1}. The process of the MFO technique consists of several steps: 1) assigning same number of female and male agents, 2) permitting the male candidate to choose the best place as *loc* for the selected task, 3) merging of male and female Mayfly at position *loc*, 4) offspring formation, and 5) closure of search process and exhibiting the output. The *MFO* method is anticipated to be similar to the mating technique and flighting characteristic of the Mayflies [37]. The male Mayfly works robustly, subsequently boosting the optimization process.

In a d-dimensional search, assume that there is the same number of male (M) and female (F) Mayflies, with the total number of agents (Mayflies) denoted by i = 1, 2, …, N, where N is 20. In the process of optimizing the search, each agent is initially placed arbitrarily in the search area, and allowed to move towards the best location as the iteration progresses. The male Mayfly can adjust its position and velocity to reach the best *loc*. The agent's movement towards the final target is determined by the Cartesian distance and the iteration number, like the Firefly Algorithm discussed in Ref. [38]. The equation for updating the agent's location is presented in Eq. (6):

(6) $${loc}_{i}\left(time+1\right)={loc}_{i}\left(time\right)+{velocity}_{i}\left(time+1\right)\text{,}$$

The half of the males and females perform mating and form offspring. The offsprings formed are denoted in the mathematical form in Eqs. (7), (8)).

(7) $${offspr}_{1}=P\;x\;Male+\left(1-P\right)\;x\;Female\text{,}$$

(8) $${offspr}_{2}=P\;x\;Female+\left(1-P\right)\;x\;Male\text{,}$$

**Fig. 2:**
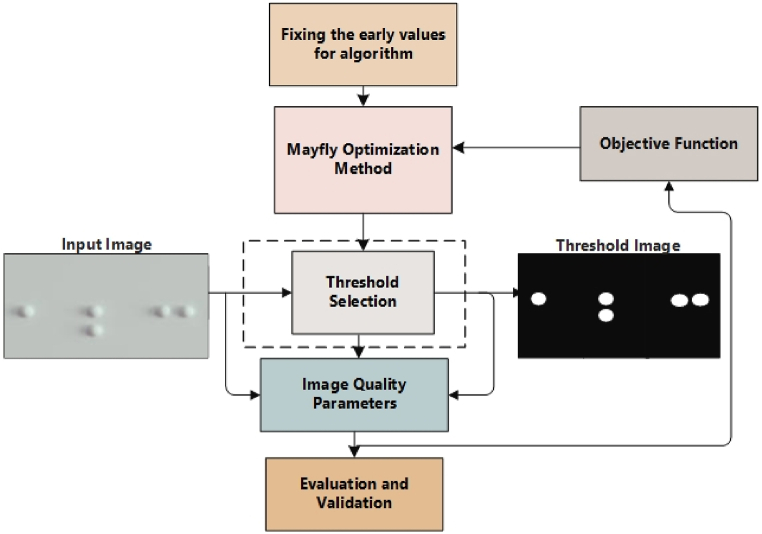
Various steps for the MFO technique.

Here, *P* presents the arbitrary values for Gauss distribution. The process of the employed thresholding method is shown in Fig. 2.

**Fig. 3:**
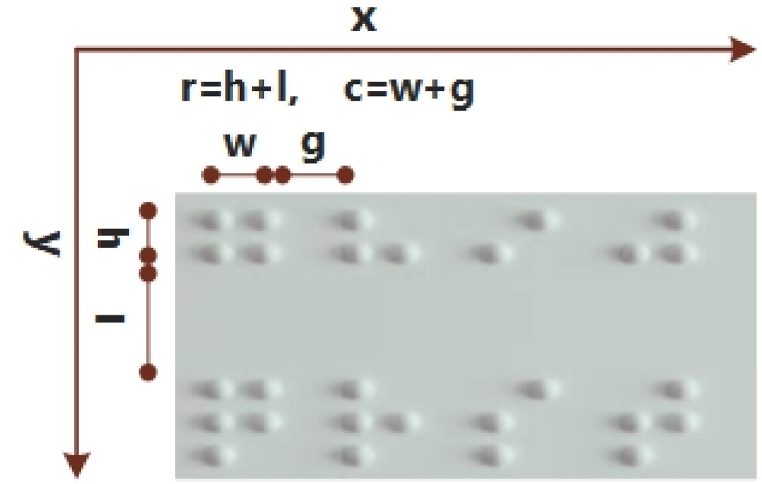
Distance estimation among braille cells.

### 3.2 Cropping

In this phase, we utilized the character positioning cropping algorithm (CPCA) method to obtain braille cells from the segmented images. It considers the first and last location of the braille dots in the segmented regions attained from the preceding step. The two braille dots are presented by (*x*_min_*, y*_min_) and (*x*_max_*, y*_max_). Furthermore, the following steps are employed for cropping the braille cells.

1. Define the total columns and rows in the region of interest in segmented cells. In the below equations, r is the sum of the height of one braille dot and the vertical distance from the braille dots in the next row. Whereas, c is the sum of the width of the individual braille symbol and the gap from the next braille symbol in the same row, as shown in Fig. 3. The total rows and columns are computed as in Eqs. (9), (10)).

(9) $$N_{rows}=\frac{y_{\max}-y_{\min}}{r}\text{,}$$

(10) $$N_{cols}=\frac{x_{\max}-x_{\min}}{c}\text{,}$$

2. Determine the coordinates using the row and column numbers for any cell as computed in Eqs. (11), (12)).

(11) $$i=y_{\min}+\left(N_{rows}-1\right)\;x\;r\text{,}$$

(12) $$j=x_{\min}+\left(N_{cols}-1\right)\;x\;c\text{,}$$

3. In the end, we used the coordinates attained in the 2nd step for cropping the braille cells from the image and utilized them in the next stage of the proposed technique.

The distance among braille cells is measured as shown in Fig. 3.

### 3.3 LeNet-5 for braille cell recognition

**Fig. 4:**
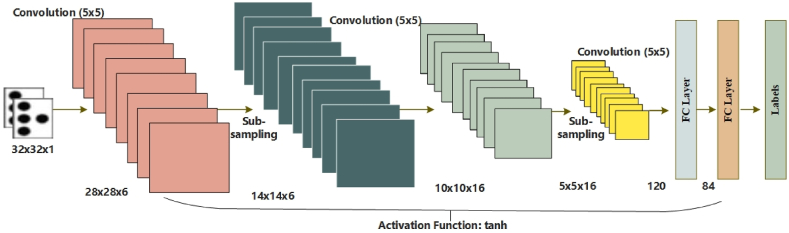
Architecture of LeNet-5.

In this step, the braille cells are recognized from Section 3.2, and the patterns are learned by the LeNet-5 deep learning model [39]. The advantage of using the deep learning (DL) technique is an automated feature extraction process for classification [34,35]. Moreover, the DL models achieve higher accuracy than traditional ML methods for computer vision tasks [40]. The structure of LeNet-5 is shown in Fig. 4. LeNet-5 is a deep neural network architecture that consists of 7 layers, including 3 convolutional layers, 2 pooling layers, and 2 fully connected layers. The architecture was specifically designed for handwritten digit recognition on the MNIST dataset, but it has since been applied to various image classification tasks.

**Fig. 5:**
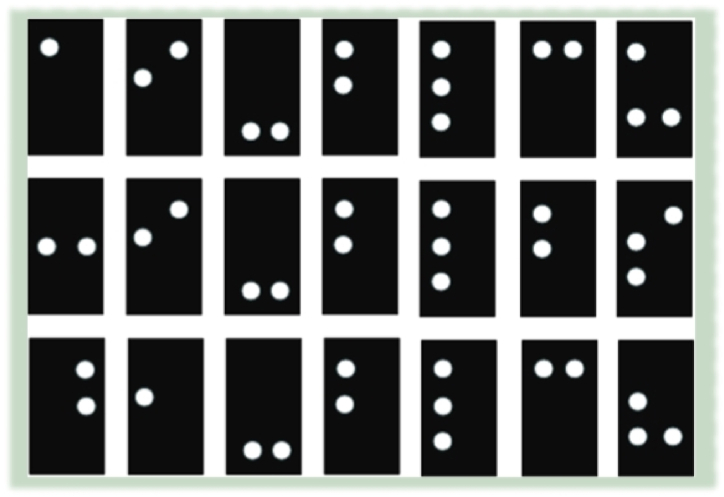
Some segmented and cropped braille cells.

**Fig. 6:**
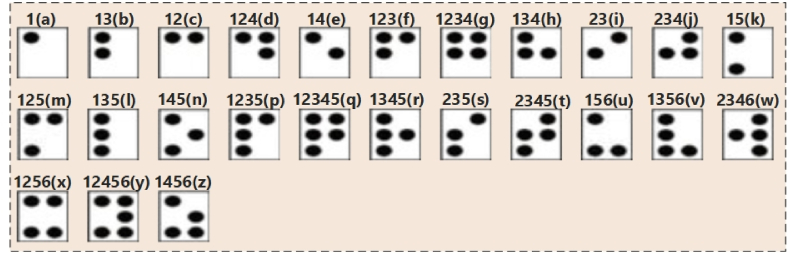
Braille cells from Dataset-1 with class labels corresponding to alphabets.

After the segmentation, we obtain the segmented braille dots, as shown in Fig. 5. We trained the LeNet-5 using the binary images of braille cells, as shown in Fig. 6. The model learned the features from the cropped braille cells according to the labels. The braille image labels indicate numbers that correspond to various dot combinations within the braille cells. To train the deep convolutional neural network (DCNN), these labels are transformed into a binary format using the one-hot encoding method. This is a widely used approach for representing categorical variables as binary vectors, where each element in the vector represents a possible category.

During the testing and validation phase, the output of the LeNet-5 model is a probability distribution over the possible categories. To obtain the final predicted label, the category with the highest probability is selected as the predicted label. This predicted label is then converted back to its corresponding digit representation for evaluation and reporting of results. Overall, the process involves converting the braille digit labels to binary format for training the model and converting the predicted binary labels back to digit format for evaluation and reporting.

One of the advantages of LeNet-5 is its ability to deal with small datasets and input image sizes [41]. This makes it suitable for tasks with limited data, such as medical image analysis. Additionally, the architecture is relatively simple compared to more recent CNNs, which makes it computationally efficient and easier to implement. In our work, we compared LeNet-5 with two other popular CNNs, VGG16 and ResNet, and found that LeNet-5 performed the best. However, it is worth noting that the outcome of a CNN can depend on various factors, including the specific task, dataset, and implementation details.

This architecture was originally designed for the MNIST dataset, which comprises 28 × 28 pixel samples of handwritten digits, but it has been adapted to work with the above input image size of 32 × 32 pixels. The output layer of the LeNet-5 in our model is a dense layer with several nodes, which corresponds to the output labels classification task. During training, the weights and biases of the LeNet-5 are learned by minimizing the cross-entropy loss function between the predicted and ground truth labels. This is a standard approach for training neural networks, where the goal is to find the set of weights and biases that minimize the difference between the predicted and actual outputs. The optimization process is typically performed using gradient descent or one of its variants.

The LeNet-5 model is trained to utilize the Adam optimizer, having a learning rate of 0.001, a momentum of 0.9, and a batch size of 32. Adam is a popular optimization method that combines ideas from both momentum and RMSProp to perform adaptive gradient descent. It is known as working well in a wide range of domains and is commonly used in deep learning. The number of epochs is set to 100, which specifies the number of times the entire training dataset is passed through the model during training. This is a hyperparameter that needs to be tuned carefully to balance between overfitting and underfitting. In our work, 100 epochs seem to have provided promising results. The dataset is randomly split into 80% for training and 20% for testing. This is a standard practice in machine learning to evaluate the model's performance on unseen data. The goal is to ensure the model generalizes well and does not overfit the training data.

### 3.4 Mapping

In this phase, the categorized labels attained from the above step are transformed into digits comprising the various patterns of braille dots. The labels were produced in the preceding step depending on the input cells following the LeNet-5 architecture. These categorized labels map the corresponding characters specific to language searching in the lookup table. The lookup table consists of numerous digits comprising several groupings of braille dots ranging from “Nill” to “all six dots raised” with their respective characters specific to the linguistic. Some of the combinations, along with the characters of the linguistic (Arabic and English), are exhibited in Table 1. Further, a subdivision of dots, along with class labels of languages such as Arabic, English, Hindi, and Bangla numbers, is reported in Table 2.

**Table 1:**
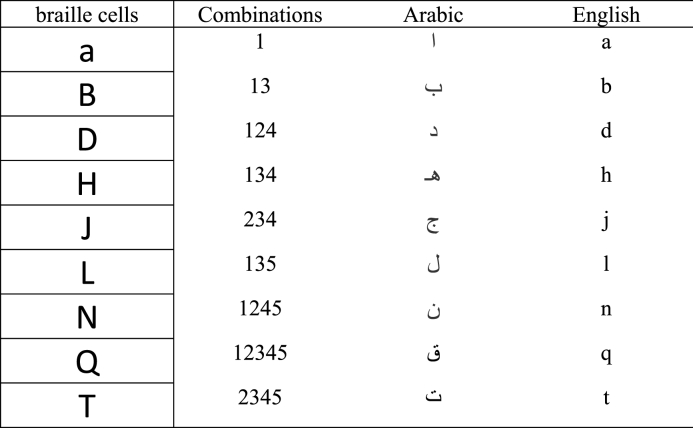
Some braille dots combinations with corresponding language characters.

**Table 2:** Braille dots combinations and their respective numbers according to four languages.

Braille cells	braille Dots Combinations	Numbers
		(Arabic, English, Hindi, Bangla)
	
#j	2456 234	0
#a	2456 1	1
#b	2456 13	2
#c	2456 12	3
#d	2456 124	4
#e	2456 14	5
#f	2456 123	6
#g	2456 1234	7
#h	2456 134	8
#i	2456 23	9

The outcome of this final phase is a natural language text that consists of the matching characters of categorized braille cells. Each braille cell consists of 6 dots arranged in a specific pattern, which can be interpreted as a specific character. For example, the letter “a” is represented by the first dot in the first column of the braille cell, while the letter “b” is represented by the first two dots in the first column of the braille cell.

## 4 Experimental evaluation

The section describes the methods used to analyze the suggested model's performance. It also gives employment details, procedures for training and testing, and numerous experiments.

### 4.1 Datasets

**Fig. 7:**
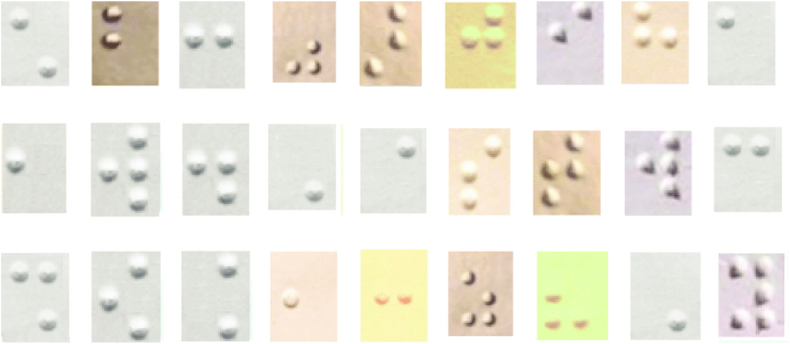
Braille cell images from the collected Dataset [42].

In this segment, two braille image datasets were employed to assess the convolutional neural network (CNN) model of the proposed methodology. The first dataset [41] comprises 1404 braille cell images labeled and representing 27 symbols corresponding to alphabet characters. The images were gathered from the web, signs, and rare books and were deliberately altered to increase the difficulty level. Each braille cell in the images was independently cropped and labeled. The second dataset [6] comprises 5420 labeled samples of 37 braille symbols representing alphabets, numbers, and punctuation. The images in this dataset were also sourced from the web, symbols, and rare books and were purposely altered to include noise. The braille cells in the images were cropped and labeled independently, similar to the first dataset. Both datasets were likely selected based on their diversity and representativeness of the symbols they contain, as well as their similarity to real-world braille images that the proposed approach would need to classify. Each dataset was split into three subsets: train, test, and validation. 60% of the data was utilized for training, 20% was used for validation, and 20% for testing. More specifically, from Dataset-1, 1124 samples were used for training and 280 samples for testing. A testing dataset consisted of varying samples, including the images that had braille on both sides. A space character was also included in the dataset to allow the identification of individual words in a string. Some samples collected from the datasets are shown in Fig. 7.

Moreover, from Dataset-2, 2000 samples were used for cross-validation. Some samples of braille cells corresponding to class labels from Dataset-1 are shown in Fig. 6. Note that the class labels (the numbers above the cell) are different from the conventional dot numbering in the braille cell. The conventional dot numbering is as follows: The braille cell involves six dots ordered in 2 columns and 3 rows. Each dot has numbers from 1 to 6. Dot 1 is in the top left corner of the cell. Moving below to the middle row is dot 2, dot 3 is in the left bottom, and so on for the right column.

### 4.2 Metrics

Precision, Accuracy, Recall, and F1 score were used to assess the proposed system. The equations are presented as Eqs. (13), (14), (15), (16), respectively.

(13) $$\text{Precision}=\frac{\text{TP}}{\text{TP}+\text{FP},}$$

(14) $$\text{Accuracy}=\frac{\text{TP}+\text{TN}}{\text{TP}+\text{TN}+\text{FP}+\text{FN}}\text{,}$$

(15) $$\text{Recall}=\frac{\text{TP}}{\text{TP}+\text{FN}}\text{,}$$

(16) $$F1\;\text{score}=2*\;\frac{\text{Precision}*\text{Recall}}{\text{Precision}+\text{Recall}}\text{,}$$

In classification problems, the accuracy metric is widely used as an evaluation metric, which indicates the percentage of correctly classified instances in the dataset. Additionally, precision measures the percentage of true positive estimates among all positive likelihoods. It is beneficial when the damage due to false positives is high, as in medical imaging. Conversely, recall computes the percentage of true positives among all actual positive cases and is useful when the expense of false negatives is high, as in fraud detection. Moreover, the F1-score, the harmonic mean of precision and recall, provides a stable evaluation of the classifier's performance.

### 4.3 Results

The proposed model takes braille image as input and then extracts braille cells to transform them into a sentence. It achieved high accuracy on both Dataset-1 and Dataset-2, with an accuracy of 99.77% on the test instances of Dataset-1 and 99.80% on the test instances of Dataset-2. The outcomes indicate that our approach is effective and robust in classifying braille cells accurately.

On the other side, we cross-validated the results using the training set of Dataset1 and 1000 samples from Dataset2. We attained 98.1% accuracy and 98.7% F1 score for cross-validation. The outcomes indicate that our approach is effective and robust in classifying braille cells accurately.

Apart from assessing the accuracy, the model's performance was also evaluated based on the F1-score, a weighted average of precision and recall. The F1-score provides a more balanced assessment of the model's performance by considering both false positives and false negatives. The proposed method achieved F1-scores of 99.78% and 99.21% on the test samples of Dataset-1 and Dataset-2, respectively. Additionally, the proposed approach achieved a precision of 99.77% on Dataset-1 and 98.67% on Dataset-2, whereas the recall for Dataset-1 was 99.79% and for Dataset-2 was 99.75%.

These high results in accuracy, precision, recall, and F1-score suggest that the proposed approach effectively classifies a range of braille symbols and images and can potentially be used in real-world applications. However, it is important to note that the evaluation was accompanied on a limited set of datasets. As a result, the proposed approach's performance may vary when applied to different datasets or real-world scenarios. Therefore, further evaluation and testing are necessary to determine the robustness and generalizability of the proposed approach.

The results shown in Tables 3 and 4, and the comparative analysis in Table 5, demonstrate that the suggested model outperforms both the OBR model and the model proposed in recent works. The model's improved performance is highlighted in boldface font, indicating that the differences in accuracy and F1-score are statistically significant. However, it is necessary to note that the suggested approach still has limitations that must be addressed in future work. In addition, the proposed method has some limitations that should be considered. For instance, the multilingual mapping step does not include braille abbreviations and contractions, which may restrict its usefulness in certain situations. Moreover, the method cannot automatically identify the language of the braille document, and we need to inform a system manually, which could pose a significant challenge in multilingual contexts.

**Table 3:** Performance of *Fly-LeNet* on test samples of Dataset-1.

Class label	Accuracy (%)	Precision (%)	Recall (%)	F1-Score
1	100	100	100	100
12	100	100	100	100
13	100	100	100	100
124	99	98.7	99	98.85
123	100	100	100	100
1234	99	99	98	98.50
14	100	100	100	100
134	100	100	100	100
234	100	100	100	100
15	100	100	100	100
23	99	98	99	98.50
1245	100	100	100	100
135	100	100	100	100
125	98	98	99.5	98.74
1235	100	100	100	100
12 345	100	100	100	100
145	100	100	100	100
235	100	100	100	100
1345	100	100	100	100
156	100	100	100	100
1356	100	100	100	100
2345	100	100	100	100
0	100	100	100	100
1456	100	100	100	100
12 456	100	100	100	100
1256	100	100	100	100
2346	99	100	99	99.50
**Avg. Accuracy**	**99.77**	**99.77**	**99.79**	**99.78**

**Table 4:** Performance of *Fly-LeNet* on 2000 test samples of Dataset-2.

Class label	Accuracy	Precision	Recall	F1-Score
1	100	100	100	100
12	100	100	100	100
13	100	100	100	100
124	99	98	97.8	97.90
123	100	100	100	100
1234	100	100	100	100
14	100	100	100	100
134	100	100	100	100
234	99	98.5	99.5	99
15	100	100	100	100
23	100	100	100	100
1245	100	100	100	100
135	100	100	100	100
125	98.5	98	99.3	98.65
1235	100	100	100	100
12 345	100	100	100	100
145	100	100	100	100
235	100	100	100	100
1345	99	98.3	99.4	98.85
156	100	100	100	100
1356	100	100	100	100
2345	99	99	98	98.50
0	100	100	100	100
1456	100	100	100	100
12 456	100	100	100	100
1256	100	100	100	100
2346	100	100	100	100
3	100	100	100	100
5	100	100	100	100
6	100	100	100	100
345	100	100	100	100
356	99	98	99.3	98.65
35	100	100	100	100
56	100	100	100	100
34	100	100	100	100
236	100	100	100	100
2456	99	98	97.4	97.70
**Avg. Accuracy**	**99.80**	**98.67**	**99.75**	**99.21**

**Table 5:** Comparison with similar existing methods.

Reference	Dataset	Accuracy (%)	F1-Score
Hsu et al. [6]	Dataset 1	98.73	–
AlSalman et al. [42]	Dataset 1 Dataset 2	99.29 98.99	99.30 99.00
Li et al. [13]	DSBI BAS	– –	99.66 96.38
*Fly-LeNet*	Dataset 1 Dataset 2	99.77 99.80	99.78 99.21

Finally, the *Fly-LeNet* must be assessed on a large-scale dataset comprising braille samples for various natural languages to determine its generalizability and robustness. This will require collecting and annotating many braille images, which may be challenging and time-consuming. Nonetheless, these limitations provide further research and development opportunities to improve the proposed approach's effectiveness and expand its applicability.

**Fig. 8:**
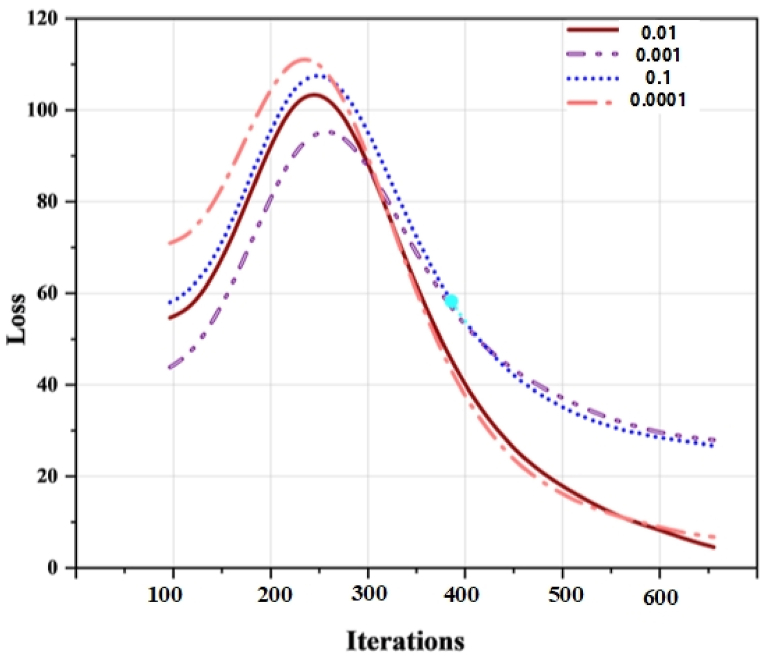
Results of changing learning rate during training on Dataset-1.

**Fig. 9:**
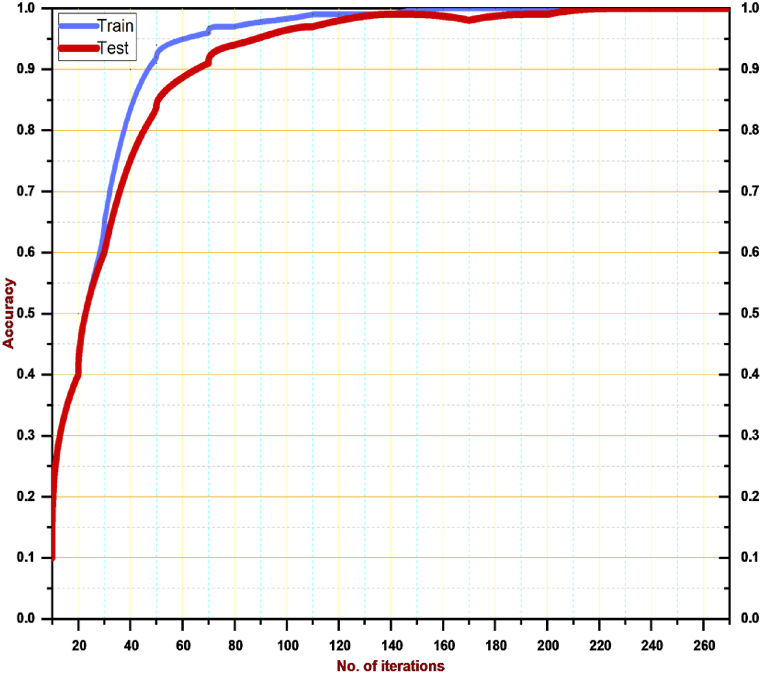
Training and Testing accuracies of *Fly-LeNet* on Dataset-1.

We experimented during the training on Dataset-1 by changing the learning rate. Fig. 8 shows the outcomes of this experiment. It is visible from the visualization that we attained the best result on loss when we used 0.01 as the learning rate. The reason could be that 0.0001 is too small to reach convergence, and 0.1 is large enough to increase the training error and slow the training process. The training accuracy of *Fly-LeNet* on Dataset-1 is shown in Fig. 9.

### 4.4 Comparison with existing methods

In this section, we compare the results of *Fly-LeNet* with the existing braille language conversion system. The statistics are shown in Table 5. We used the same datasets for this experiment and trained the models proposed by Hsu et al. [6] and AlSalman et al. [42], and it is visible from the results that *Fly-LeNet* outperforms both of these methods. Hsu et al. employed a novel approach, combining a CNN with a character extraction algorithm based on ratios (RCSA). Additionally, a fresh dataset was constructed, encompassing 26,724 braille images, each of which was meticulously labeled. This dataset encompasses 37 distinct braille symbols, equivalent to 71 unique English characters, punctuation, spanning the alphabet, and numerical digits. The input to the proposed model was braille images, and then preprocessing was performed to attain individual words. Notably, the CNN model achieved an impressive prediction accuracy of 98.73% when evaluated on the test set. In our previous work [42], we attained 99.29% and 98.99% accuracy on Dataset-1 and Dataset-2, respectively. However, for *Fly-LeNet,* accuracy has increased to 99.77% and 99.80% for Dataset-1 and Dataset-2, respectively. In Ref. [13], authors proposed a BraUNet, recognizing braille dots directly employing the UNet model. They used long-ranged feature connections to extract more low-level features from braille characters. The proposed model performed significantly well for complex dual-sided braille samples and hand-generated braille samples. More specifically, the BraUNet model receives input of the whole braille page and then extracts the local features using UNet. Then, it post-processes the features and detects the braille characters in the entire document. The authors utilized two datasets for experiments such as DSBI and BAS, achieving the maximum F1 score 99.66% and 96.38%, respectively.

The input to our previous [42] and current work is a single braille document as images. From the comparative analysis, it is clearly noticeable that the proposed model is more efficient in terms of accuracy and F1 score for the recognition and conversion of braille words to the corresponding language. The strength of the proposed model is due to the adaptability, simplicity, and robustness of the utilized Mayfly optimization technique for accurate braille dots segmentation. Moreover, the architecture of the proposed *Fly-LeNet* was initially designed for handwritten digit identification. Therefore, *Fly-LeNet* played a crucial role in the braille recognition process using minimal computational resources due to its simplest structure. Therefore, our proposed model, *Fly-LeNet,* is more capable and effective in transforming the braille document into any natural language.

### 4.5 Performance of segmentation technique

This experiment evaluated our proposed segmentation approach based on the metrics: DOI, TC, area, and no. of pixels. The mathematical forms for these parameters are provided in Eqs. (17), (18), (19).

(17) DOI=ω1+ω2,

(18) TC=∑x=1m∑y=1n(I′∩I)∑x=1m∑y=1n(I′∪I),

(19) $$area=\sum_{i=1}^{m}\sum_{j=1}^{n}I\left(i,j\right)\text{,}$$

Our segmentation approach was assessed by comparing the outcomes on 40 samples from each dataset, with TC values ranging from 0 to 1. The metrics used to compute the results were based on the corresponding ground truth images. Table 6 reports the outcomes of Dataset-1, specifically for 9 braille images, and the outcomes exhibit the effectiveness of the segmentation technique we selected. The area refers to the size or extent of a segmented region by our proposed model.

**Table 6:** The segmentation results for some samples from Dataset-1.

Braille Cells	DOI	TC	Area (nm^2^)
a	0.99	0.98	0.2 × 10^14^
b	0.98	0.98	1.3 × 10^13^
d	0.98	0.99	1.8 × 10^12^
h	0.99	0.96	1.3 × 10^14^
j	0.99	0.99	2.4 × 10^12^
l	0.98	0.97	2.8 × 10^13^
n	0.98	0.98	2.8 × 10^11^
q	0.99	0.98	2.1 × 10^13^
t	0.99	0.98	2.4 × 10^13^

## 5 Conclusion

To enable communication between visually impaired individuals and those who can see, a deep learning method named *Fly-LeNet* has been suggested to transform braille images into text in multiple languages. The process involves various stages, including obtaining and processing braille images, identifying and extracting braille cells, recognizing them, and mapping them to multilingual characters. First, we gathered the braille images and segmented the region of interest using the mayfly optimization approach along with Kapur's thresholding method. Then, the braille cells are extracted using the character positioning cropping algorithm (CPCA) based on standard measurements. Finally, a DL model, LeNet-5, is trained to recognize the cells and retrieve corresponding labels from a lookup table to retrieve multilingual characters. The DL model's performance was assessed through experiments on two braille image datasets, which showed that the proposed method had a classification accuracy of 99.77% and 99.80%, respectively, outperforming existing models. Furthermore, the architecture of LeNet-5 is less complex than some of the latest DL algorithms like DenseNet. Moreover, after fine-tuning and training, the proposed system can be used for any natural language.

The biggest challenge we faced during the development of our system was the unavailability of datasets for most foreign languages. Due to this, we performed experiments using braille images rather than braille documents. Therefore, in the future, we aim to gather enough foreign language datasets to be used for training and aiding visually impaired people from developing countries. Moreover, we aim to modify our system to detect the source language automatically by identifying the common words in the language. For example, in English, ‘a' and ‘the' are common words in almost every sentence.

## Funding statement

This project was funded by the 10.13039/501100005725National Plan for Science, Technology and Innovation (MAARIFAH), King Abdulaziz City for Science and Technology, Kingdom of Saudi Arabia, Award Number (5-18-03-001-0004).

## Data availability statement

Data sharing does not apply to this article as authors have used publicly available datasets, whose details are included in the “experimental results and discussions” section of this article. Please contact the authors for further requests.

## CRediT authorship contribution statement

**Abdulmalik Al-Salman:** Writing – review & editing, Writing – original draft, Supervision, Software, Project administration, Methodology, Investigation, Funding acquisition, Formal analysis, Conceptualization. **Amani AlSalman:** Writing – review & editing, Visualization, Validation, Resources, Methodology, Data curation, Conceptualization.

## Declaration of competing interest

The authors declare that they have no known competing financial interests or personal relationships that could have appeared to influence the work reported in this paper.
